# The role of feedback in supporting trainees who underperform in clinical environments

**DOI:** 10.3389/fmed.2023.1121602

**Published:** 2023-04-25

**Authors:** Rola Ajjawi, Margaret Bearman, Elizabeth Molloy, Christy Noble

**Affiliations:** ^1^Centre for Research in Assessment and Digital Learning, Deakin University, Melbourne, VIC, Australia; ^2^Department of Medical Education, Faculty of Medicine, Dentistry and Health Sciences, University of Melbourne, Melbourne, VIC, Australia; ^3^Academy for Medical Education, Medical School, The University of Queensland, Herston, QLD, Australia

**Keywords:** feedback, failure, underperformance of learners, health settings, learning culture, motivation

## Abstract

**Introduction:**

Underperformance in clinical environments can be costly and emotional for all stakeholders. Feedback is an important pedagogical strategy for working with underperformance – both formal and informal strategies can make a difference. Feedback is a typical feature of remediation programs, and yet there is little consensus on how feedback should unfold in the context of underperformance.

**Methods:**

This narrative review synthesises literature at the intersections of feedback and underperformance in clinical environments where service, learning and safety need to be considered. We do so with a critical eye towards generating insights for working with underperformance in the clinical environment.

**Synthesis and discussion:**

There are compounding and multi-level factors that contribute to underperformance and subsequent failure. This complexity overwrites simplistic notions of ‘earned’ failure through individual traits and deficit. Working with such complexity requires feedback that goes beyond educator input or ‘telling’. When we shift beyond feedback as input to process, we recognise that these processes are fundamentally relational, where trust and safety are necessary for trainees to share their weaknesses and doubts. Emotions are always present and they signal action. Feedback literacy might help us consider how to engage trainees with feedback so that they take an active (autonomous) role in developing their evaluative judgements. Finally, feedback cultures can be influential and take effort to shift if at all. A key mechanism running through all these considerations of feedback is enabling internal motivation, and creating conditions for trainees to feel relatedness, competence and autonomy. Broadening our perceptions of feedback, beyond telling, might help create environments for learning to flourish.

## 1. Introduction

### 1.1. The problem of underperformance in health professions education

Underperformance and subsequent failure are critical experiences that can have many damaging effects. For trainees, failure can be financially costly, involving reputational loss, and emotional stress, shame and stigma (1, 2); for teachers, it is a time drain, leading to ambivalence, anger, stress and guilt (3, 4); and for the clinical environments, there is potential for compromise of patient safety, financial loss, litigation and lost productivity (5, 6). While remediation models are diverse (7) feedback features in all attempts to work with underperformance (8–12) and is the focal point for this narrative review.

Remediation is the common course of action for an episode of underperformance, or a series of events that suggest a pattern of underperformance – facilitating a ‘correction’ for trainees who have moved off course (13). This aligns to the notion that there is a single linear course to be followed for ‘able’ trainees. Ellaway et al. (5) differentiate between remedial action and remediation. They define remedial action as “largely supportive, informal, and short-term events … in which a preceptor facilitates a learner’s progression toward professional mastery and independent practice” (p. 394). Remediation on the other hand, “is a formal response to sustained underperformance, with a different schema (its collected rules, roles, responsibilities, and thresholds) than the ones for the mainstream curriculum” (p. 394). The depth of the intervention is tailored to the persistence and scope of the trainees’ difficulties. Clearly, feedback can play a role in both these pedagogical responses – whether through formal feedback-on-action interventions including coaching and/or more embedded and informal forms of feedback-in-action (14, 15).

For this paper, we conducted a narrative review to synthesise how feedback can be used to support students who underperform in clinical environments. Narrative reviews generally do not rely on systematised search strategies, and they enable a wide range of papers to be included to provide interpretation and critique (16). Our search included papers where feedback and/or underperformance were the primary focus in medical and higher education literature, aligning with our expertise. The paper follows in the tradition of narrative reviews where we develop themes for how feedback might be understood and employed in the context of underperformance.

We start from the perspective that underperformance and learning are inextricably intertwined. Feedback is defined as a social process where trainees have an active part to play in making sense of performance relevant information, within social, cultural, relational and material environments (17). We use the term trainee to represent learners at all stages of their journey: from pre-service students undertaking clinical placements to clinicians completing specialised training. We primarily focus here on sustained underperformance, although sometimes failure might occur at a micro-level, e.g., where trainees are allowed to fail without compromising patient safety (18), or as a self-assessment where performance was below personal expectation (2).

A common response to underperformance is that supervisors do more – observation and feedback – to help trainees. However supervisors report doing more of the same to little effect (3). So, if telling trainees what to do is not the answer, how might feedback be used to support those who are underperforming in clinical environments? Overall, we aim to challenge the perception that more telling is better. We start with examining the language of struggle, failure and underperformance critiquing the discourses of deficit, meritocracy and linear achievement. We then synthesise feedback literature relevant to working with trainee underperformance. Through critiquing a broad range of literature (health professions, higher education, organisational psychology), we discuss how dominant feedback trends might help with underperformance and consider where these might not be so helpful within complex clinical environments. The paper ends with insights for clinical educators about the role of feedback in working with underperformance.

## 2. The deficit discourses of feedback – what’s in a name?

*Struggling*, *underperforming*, *failing* or even the *problem* student/trainee can be found in health professions education literature. Meritocratic discourses of education present a rigid and linear notion of potential which prescribe fixed, developmental timeframes – veiled in “an aura of objectivity” that obscures the role of privilege, hierarchy, class, and power (19). Any deviance from these developmental timeframes constructs a perception that trainees have failed to engage with education processes in an idealised manner and where individual deficit can be ‘fixed’ through remedial forms of support (20). Failure and underperformance are thus attributed to the individual – as resulting from a trainee’s individual traits or acts that “earn them failure” (21). The deficit discourse of failure serves to position struggling trainees as other – a “not like us” position (4) which only compounds that individual’s struggles (7). This ‘othering’ can also exclude trainees from learning opportunities and has implications for how underperforming trainees might engage with feedback.

The discourses of performance, underperformance, achievement and failure are much more complex than is often represented. For example, grades may reflect structural or institutional bias (22). Failure discourses can be gendered with the ideal successful learner constructed as male, white, middle class and able-bodied, an autonomous individual unencumbered by domestic responsibilities, poverty, or self-doubt (23). Women in certain academic disciplines may “already feel that they can never be good enough or never get it right … and this is not, we suggest, about individual failings of confidence, but a result of the systematic positioning of some groups in our society as of less worth or value than others” (23) (p. 609). This is particularly true in male dominated specialties such as surgery (24). Intersecting with gender, is class, where working-class students are less likely to seek help compared with those from a middle-class background (25), with such patterns of behaviour attributed to socialised logics of action carried over from schooling (26). The persistent differential attainment between white and ethnic minority cohorts in medical education suggests that progression has less to do with capability and more to do with context and race (22). Indeed standards themselves, against which we judge performance are sociocultural and material constructions (27).

Research in higher education shows that failure and underperformance are multifactorial due to an interplay of dispositional, situational, relational and cultural factors (1). Dispositional factors include the ways trainees approach their study, what they prioritise, how they self- and effort-regulate. Situational factors – factors outside an individual’s control – might arise that influence trainees’ ability to study and curtail their efforts such as illness (their own or a loved one). Relational factors include feelings of isolation, exclusion and marginalisation where trainees are afraid of asking for help due to stigma or repercussions. Finally, cultural factors can impede progress where the pedagogical and pastoral support mechanisms available in a particular location are not suitable for the particular trainee. This is illustrated by the phenomenon where a trainee cannot seem to overcome a label of underperformance in a particular rotation and yet they flourish in another environment (6).

This complex confluence of factors for trainees who are underperforming demands a multi-faceted approach to remediation. However, with this broader conceptualisation of underperformance, the band of influence of feedback may be quite narrow, or even, counter-productive in certain situations. This counters the ‘face value’ of feedback as an always valuable means for working with underperformance: this response suggests that the bigger picture is important in charting ways of thinking about feedback in clinical environments.

What this focused summary shows is the complexity of how underperformance is constructed, not just through individual acts but through systems of disadvantage that go beyond the individual. Much like the challenge with the ubiquity of the language of ‘giving feedback’; the language of the ‘underperforming trainee’ comes with its own baggage. We have tried in the language of the paper, not to link underperformance to individual attributes or traits but to acknowledge the social, cultural, historical and material constitution of (under)performances. We also draw on our previous work, to remind that feedback rules do not work in the face of such diversity and complexity (28), instead we offer educational principles derived from the literature for individuals to use to reflect on and inform their practices.

## 3. Feedback and underperformance

While much has been written about the power of feedback for learning (29, 30), less is known about the role of feedback in supporting trainees who are failing to meet performance expectations. We outline some of the insights from the broader education literature. A recent realist review of feedback in higher education (31) identifies that undergraduate students with differential achievement approach feedback differently. For example, negative feedback comments might spur some students to greater effort regulation but in students with pre-existing perceptions of low self-efficacy it can lead to demotivation and unhelpful feedback behaviours, such as avoiding seeking clarification from supervisors and less action on feedback comments.

The review identified Ryan and Deci’s (32) self-determination theory (SDT) as providing a strong theoretical explanation for feedback interventions that work in open-ended written university assessment. SDT, with psychological roots and strong evidence, suggests that internal motivation is leveraged through conditions that foster an individual’s perceptions of relatedness, competence and autonomy (32). A realist review of remediation also identified motivation as a key mechanism for effectiveness (9). It has been proposed that SDT explains why students who underperform appear so disengaged and to lack insight (10): we can conjecture that being labelled as underperforming or failing can be a threat to perceptions of competence as well as potentially rupturing perceptions of relationality and that this can interfere with feedback practices. We also know that remediation can act as a threat to autonomy – at least for physicians (7). The follow-on effect would be loss of internally driven motivation. Precisely the time when you want trainees to remain motivated and engaged!

## 4. Troubling the intersections of feedback and underperformance in clinical environments

Against a backdrop of SDT, we present themes for how feedback conversations might be positioned to support trainee learning in the context of underperformance. Relationships are described as the backbone of feedback (33) and form a necessary pillar of SDT. Emotions are central to feedback and relationality and so we wrangle with these next. Feedback literacy opens perspectives into perceptions of competence and autonomy. We end by discussing feedback cultures as these are where conditions for motivation, performance and engagement play out.

### 4.1. Relationships, trust and shadow systems

Feedback in clinical environments is strongly mediated by relationships (34). In their research, Telio et al. (35) found that the strength of the educational alliance as perceived by trainees influenced how trainees made sense of and used feedback information. By recognising the important role of relationships in feedback effectiveness, we disrupt the focus on the message with typically linear thinking of positive valence comments leading to positive reactions (and vice versa). Instead, perceptions of the strength of the educational alliance might be a better predictor of feedback effectiveness. It is hard to form a strong educational alliance if you feel victimised or othered.

Supervisors have reported that they find it challenging to have feedback conversations with trainees when the observed activity is seen to be below-par, or if a series of observed events have been viewed to be sub-standard (36). Supervisors not only report the emotional burden they feel after engaging in these discussions (37), but observational studies of feedback show that supervisor language in these conversations takes on a distinctive tenor – what some have reported as “vanishing” (38), others as “mealy mouthed” (39), and others as “hesitant and apologetic.” Johnson and Molloy (40) describe how supervisors talk in circles in an attempt to diminish the negative emotional impact on learners. What is not clear is the extent to which this fear of upsetting trainees will be fulfilled. We can only imagine that such mealy-mouthed approaches do not provide trainees with great confidence that their educators believe they are capable of engaging in candid discussion about their performance. The work of Castanelli et al. (41) in the anaesthesia supervisory context suggests that performance conversations are seen to be easier and more effective if the trainee trusts in the supervisor. Talking in circles or avoidant approaches may indicate a lack of bi-directional trust.

When a trainee has low trust in the supervisor, there are several strategies they might employ when it comes to feedback. Firstly within the relationship, the trainee may attempt to reduce what they reveal to the supervisor, both in terms of working alongside the supervisor, but also what they choose to share in feedback conversations (41). “Trainees learn to expose their authentic practice in assessments as an expression of trust” (p. 288) which has implications for the complexity of challenge they might take on in front of the supervisor, and the degree of candour they display when talking about their practice struggles in the context of feedback or assessment conversations. These findings did not suggest that ‘poorly performing students’ hide, or that they ‘game’ feedback conversations, but rather that trainees, regardless of their ‘performance status’ made judgements about the extent to which their supervisors were trustworthy (demonstrated through action, and occasionally by word of mouth or status). The judgements of trust were seen to influence what trainees were willing to share, how curious the trainees were within working and educational encounters (most often intertwined) and how trainees sought out opportunities for alternative feedback conversations in their environments.

Where it becomes more challenging, is when the informal networks of assessment – what Castanelli et al. (42) termed a ‘shadow system’ – operates to forewarn clinicians that a poorly performing trainee lurks amongst them. One supervisor described making trainee performance judgements based on “lots of interactions, phone calls, conversations … You get a bit of a vibe about it … there’s far more than just individual cases and individual procedures. It is a global assessment that is far more telling” (42) (p. 140). Thus, the trainee who may try to seek alternative viewpoints and interactions (perhaps even those that only reinforce their world view); they may be working within a system that has already deemed them ‘underperforming’, thereby limiting access to work opportunities that may stretch them appropriately or that might create a productive network to further support their learning in the workplace.

### 4.2. Emotions

There is a large body of literature reporting on the intersection of feedback and emotion in higher education [e.g., (31, 43)] and health professions education [e.g., (44, 45)]. Increasingly there is an acknowledgement that emotions can spur action from feedback processes, and that emotions can be an internal source of feedback in their own right. We look at each of these ideas in turn: the hurtful nature of some feedback encounters; the relationship between trainee insight and emotion; how negative emotions can be productive; and thinking about productive and caring feedback interactions.

The dominant discourse of feedback literature is that negative emotions are ‘stirred up’ by the feedback process, particularly when the conversations may challenge trainee perceptions of the worth of their work, or by extension, self (45). This is useful in some ways, as it helps with understanding some of the distress trainees feel. Moreover, the emotional legacy can extend far beyond the initial encounters that created turbulence (46). If previous feedback experiences – or feedback histories – have been harmful or damaging, it is unsurprising if a trainee avoids future feedback encounters unless their perceptions of the educational alliance and feedback culture can overcome the legacy of harmful feedback. Not only is feedback emotional business, so is failing. Research shows that far from having earned their failure through laziness or ambivalence, students cared deeply about failing and reported emotions of shame, shock and embarrassment (1). Similarly the nursing literature speaks to students’ surprise, distress, anger and self-disappointment (47). Bynum and colleagues (2) describe medical residents’ feelings of shame at fleeting moments of underperformance. Failure involves emotional work by trainees and supervisors.

Trainee surprise or shock can be seen as a failure of feedback rather than a consequence of feedback. Unless there has been a major sentinel event trainees should not be shocked if they fail a rotation or clinical placement. With good feedback processes in place, trainees should have a clear sense of progress against declarative competencies. In this way feedback builds trainees’ evaluative judgement – defined as “the capability to make judgements about the quality of work of self and others” (48) (p. 467). Developing trainees’ evaluative judgement of their clinical practice is one of the purposes of feedback that develops independent practice (49). Here, feedback processes should help trainees come to know what good quality looks like through comparisons of judgements of practice that help trainees generate their own feedback information. Pedagogies such as self-assessment, observation and peer review, feedback dialogue about performance, engagement with standards can all play a role. Thus, when trainees are surprised about failing, they have not sufficiently understood or engaged with the standards and thus lack insight into the gaps in their performance relative to the standard.

Categorising emotions as positive or negative does not equate to their effect on learning. While shame, anger, frustration, embarrassment etc. may lead to disengagement (often described as silence/quiet) from feedback where trainees may want feedback but fear disconfirming information (50). These emotions may also spur action in some trainees. Alternatively, emotions such as relief might prompt a lack of striving. The valence of the feedback comments (positive or negative) also does not equate to their effect on emotions, because relationship act as a mediator. For example, a supervisor opened a feedback interaction with ‘you did it all wrong’ – and while this might be classified as a negative comment, the trainee reported that due to the strength of the educational alliance with his supervisor he took this on board and it resulted in improvement (35).

Confidence, care and trust are three guiding emotions for productive feedback encounters (45). Underperformance in the clinical environment may compromise these – for example a trainee who has been labelled as underperforming and exposed to increased scrutiny might lose confidence in themselves. They might feel that the learning environment is more about surveillance and gatekeeping than care; this can fracture trust and an educational alliance. These emotions implicate the supervisor and trainee in needing to act together to rectify the learning environment. Actively helping trainees to reflect on and share their emotions related to feedback processes might disrupt the negative de-motivational spiral. “Emotions give feedback meaning, weight and intensity—deepening the effects of feedback and learning. If feedback provides information that indicates individuals are not who they think they are or want to be, they feel unpleasant social emotions that, if given time and reflected on in a structured way and safe environment, can prompt productive action” (45) (p. 484).

### 4.3. Feedback literacy and feedback seeking

The previous two sections have dealt with trainees’ perceptions of relatedness, and now we move to trends that support perceptions of competence and autonomy. With feedback being reconceptualised as a dynamic and social process, trainees are being positioned to actively seek and use feedback information to enhance their performance and learning. Reconceptualising feedback in this way has illuminated fresh ways to enhance feedback – beyond doing more of the same – and offers the potential to address gnarly situations where feedback is not working.

Firstly, this reframe means that trainees are in the driver’s seat for feedback processes. Yet, effectively driving this process requires new capabilities – referred to as feedback literacy – “understandings, capacities and dispositions needed to make sense of information and use it to enhance work or learning strategies” (51) (p. 1315). Unpacking the concept of feedback literacy, a little further, feedback literate learners: (1) appreciate feedback, (2) make judgements, (3) manage affect, and (4) take action. Initial work in purposefully developing trainees’ feedback literacy offered promising results because they recognise their agentic role in feedback (52). However, participants were self-selecting and so likely high performers. But what does feedback literacy offer for trainees who are underperforming? Is it reasonable to expect these trainees to be driving feedback processes?

Further exploring feedback literacy may assist here. Firstly, appreciating feedback processes means that trainees value feedback whilst recognising they have an active role to play (51). For the trainee achieving strong results in their assessments, this might be all well and good in that their experience of feedback processes has afforded enhanced learning and performance. In the face of underperformance, the experience may be less generative and less likely to be appreciated. For instance, even if the supervisor makes conscious moves to try to create conditions promoting psychological safety (perceptions of consequences of taking interpersonal risks), hearing unwelcomed feedback information risks a shame response and disengagement from feedback processes (53). It will be hard to appreciate feedback processes with this outcome. Or what if the trainee who is underperforming finds themself in a busy clinical environment where feedback processes are not accessible or apparent? It will be a bold move for a trainee to disrupt the rhythm and flow of patient care in a clinical environment not affording opportunities for active feedback engagement (54). In clinical environments, feedback processes are not as evident nor easily accessible to newcomers (i.e., trainees) or those trainees whose sense of belonging is tenuous (55). In this instance, it will be challenging to elicit the necessary performance relevant information to enhance performance. So, here the trainee faces the challenge of not enough feedback information (46, 56).

In contrast, appreciating feedback processes and being agentic has the potential to lift engagement. Rather than experiencing feedback as something which is done to them, trainees might come to appreciate that they have an active role to play, which offers them a way forward. They can, for example, ask for clarification when the supervisor’s feedback information is not guiding them on the next steps (52). While for supervisors, rather than seeking to ‘give more feedback’, taking the time to encourage trainees’ construction of goals which guide current and future actions and conversations might ‘break the cycle’ of feedback as telling (57). However, this requires trust and for the trainee to overcome potential feelings of inadequacy and loss of autonomy.

Secondly, when trainees are underperforming it is often assumed that they are failing to accurately judge their performance – a failure of insight (58). Theoretically speaking feedback literate trainees can make judgments about the quality of their own work and others, in order to inform their own learning. An important feature is refining judgements over time to enhance the robustness of their judgements and to calibrate these judgements against those of their supervisor and expected standards. Again, the circumstances of clinical practice may not afford this calibration process (indeed it may even hamper judgements). For instance, if supervisors are constantly changing, as in shift-based supervision, trainees may receive disparate feedback information. This is a challenging scenario for trainees who then need to make sense of this disparate advice to determine what constitutes quality work. Others have identified a lack of longitudinal monitoring and fragmentation of clinical supervision experiences as challenges to supervisory judgement making (59). This highlights how the development of trainees’ evaluative judgements is constrained by system design.

Yet, developing capabilities in making judgements, as part of being feedback literate, holds promise for trainees who are underperforming. For instance, work can be done to attune trainees to the inevitability of contrasting perspectives and varied sources of performance relevant information. Pedagogical strategies can be developed to enable them to make judgements and generate internal feedback through comparisons with peers’ work (60) for example. This is unlikely to be a solo adventure for trainees. Here they would benefit from guidance from peers, near peers, and supervisors as co-pilots in driving this aspect.

### 4.4. Building a feedback rich culture to support underperformance

Building feedback literacy is an important consideration when thinking about how to work with underperformance but it does not paint the whole picture (11). The importance of context is suggested in multiple literatures. Firstly, several literature reviews suggest that program level approaches to managing underperformance are critical to remediation (8, 9). Such strategies included means of normalising and scaffolding underperformance and designing appropriate formal institutional processes (10). Secondly, the clinical feedback literature points to a wide variation of cultural practices with respect to feedback (61). Finally, the availability of learning cues and opportunities to gather information about performance can vary widely from context to context (11). Thus, without appropriate systems, supportive cultural practices and rich opportunities to demonstrate capability, even the most high-achieving trainee might struggle to gainfully engage with feedback, let alone trainees who may already be overwhelmed.

Culture is a notoriously slippery term (62). This presents strong challenges when thinking about how to engage with culture, but nonetheless to ignore culture is to ignore much of what constitutes underperformance in the first place. As Steinert (63) suggests, the first question when asked about working with underperformance is whose problem is it? It may not be the trainee who needs to engage with feedback, but in fact the context itself that is in deficit. This is amplified by the need for trainees to rotate through contexts, some of which may provide feedback rich opportunities and some of which may be damaging to trainee confidence and possibly capability. However, it is also worth noting that what one trainee may experience as a rich feedback culture, another may flounder or even be damaged.

We propose several conundrums around feedback cultures and raise possible responses. We do so by drawing on the ways that culture has been conceptualised in previous literature (64). In some instances, it may be that trainees, supervisors and university educators are working within clinical cultures that are, for all intents and purposes, fixed. This not a problem if the trainee is engaging with feedback in a highly supportive environment but can be very problematic, particularly if the trainee is seen as someone who does ‘not belong’. Supporting trainees in this situation is about increasing their literacy as outlined above, but also in ensuring that there is an external point of contact to help navigate this ‘unchanging’ culture. This could be a ‘co-pilot’, or it could be in fact a totally external feedback process, so that an external person provides comments to trainees. This has been found to be very useful in general practice training (65).

In some instances, the culture may appear to overwhelm the trainee – for example the ‘way we do things around here’ can be difficult to argue with. This can indeed be damaging for all trainees but is particularly difficult if the trainee is simply positioned as being in deficit with no way to improve. In this instance, looking to standards and expectations may be the way to help bolster trainees. These types of markers of quality can help the trainee navigate an environment that threatens to be overwhelming. This again points to evaluative judgement as a means of working with underperformance: “By focusing on evaluative judgment, the learner can look beyond the particular deficit at hand, which can breed defensiveness and other unhelpful emotions, toward how the learner can assess whether a performance is good or bad” (10) (p. 12).

Another view of culture suggests that educators can agentically adapt to the feedback culture that they find themselves within. This brings in the relational means of working with feedback: by focussing on issues such as psychological safety, educators can directly ensure that the culture is adapted to work with underperformers. Key issues here, as taken from the remediation literature, include enabling appropriate sequencing of tasks for a trainee who is underperforming and building strong trusting relationships (10). These can ensure that feedback is designed into the learning experience. A particularly useful approach may be to ensure that the trainee is given the opportunity to improve on tasks they are good at: this can normalise orientation towards improving performance over a sequence of activities without feelings of deficit (10). This may mean adapting the workplace curriculum to accommodate this strengths-based feedback approach. Once the trainee is comfortable with the processes of feedback, then they may be able to work in this way in other, more challenging arenas.

A final perspective comes from the organisational literature that culture can be conceived as something that can be changed (64); this offers a means to consider how to build a supportive feedback culture for working with trainees who underperform. This is the territory of overall systems changes: approaches to normalising failure, to professional development for educators (9, 13). For example Gingerich et al. (6) notes: “There were examples where it had become clear that the trainee perceived the extra attention as ‘being mistreated’ (S22), but the supervisors and programme interpreted it as necessary supervision and appropriate feedback. This difference of opinion commonly involved the perception that the trainee was being resistant to feedback” (p. 400). Moreover, as Bearman et al. (3) point out, supervisors often simply do not have any alternative aside from telling the learner what the problem is – there is no ‘Plan B’. We think this is a possibility for faculty development and indeed where the coaching literature may come into play. Armson et al. (66) describe joint faculty and resident development for coaching – identifying process skills, which would build a productive feedback culture – in particular: “(i) relationship building, (ii) exploring reactions to feedback, (iii) exploring understanding of feedback content, and (iv) coaching for change including development of Learning Change Plan” (p.479). In addition to this, we suggest that normalising failure is key here. It does happen that trainees do engage with feedback but fail to meet the requirements of the task. It is important in these instances that trainees do not feel that this a personal deficit or that there are no options for them. What Bellini et al. (67) call “exit ramps” – ways to leave a particular course of training – can then be built into feedback conversations, not as shameful failures but as better investments of the trainees’ time to turn their attention elsewhere.

## 5. Implications of the review for practice and research

In a realist review of remediation for doctors, Price and colleagues (9) found that developing insight and motivation were two fundamental mechanisms of success. This accords with our own body of work on feedback, and the current review, where working with underperformance requires creating conditions that motivate trainees to engage with feedback processes in order to build their feedback literacy and evaluative judgements of clinical practice (with a focus on explicating what good work looks like). This should be a joint responsibility with efforts to create a trusting and caring environment within which both formal and informal feedback practices might be enacted. Learning environments rich in performance relevant information will enable this. Performance relevant information can be interpreted from environments (through for example, the qualities of work itself, patient outcomes, comparisons of performance) as well as through conversations with others, thus decentralising the focus on feedback from supervisors (68). By openly talking about feedback practices and how things are done around here we might shift some of the stigma of feedback away from the individual to shared goals and activities. We might know when feedback is too much or not enough.

Our narrative review will alert clinical educators to the pressing need to maintain and nurture motivation in trainees who are underperforming. Moreover, based on our review, we offer the following insights to creating conditions that foster trainees’ perceptions of relationality, competence, and autonomy:

(1) Attune to diversity and to the bigger picture of underperformances. What else might be happening here beyond the individual trainee? How is power and meritocracy at play? Razack et al. (19) urges educators to create space for dialogue and questioning of the assumptions that underly meritorious work, and what behaviours are perceived and valued.
(2) When considering relationships and emotions, avoid the simple ‘bad emotions, bad outcome’ and ‘relationships will conquer all’ thinking. Despite efforts at building trust, trainees might be reticent to share their self-assessments in response to questions such as “How are you travelling?” or “What should I watch for today when you demonstrate this procedure? Silence in this scenario may be an expression of agency rather than a signal of disengagement or lack of insight. It takes insight to suspend any self-assessment that compromises survival” (69) (p.ii). It might be through creating opportunities for building competence and autonomy that trust and openness follow. Notice what emotions are sticking and circulating in the clinical environment and invite a conversation about this. It might be quite revealing.
(3) Seek to develop trainees’ feedback literacy and evaluative judgement of clinical practice as alternatives to ‘telling’ that build competence and autonomy. This shifts the focus to co-construction of goals and supporting trainees to ‘patch together’ performance relevant information about their progress through multiple opportunities for comparisons of performance, evaluation against standards and conversations with peers and others.
(4) Consider how ‘shiftable’ a feedback culture really is and what is dominant within it. However, importantly for supporting trainees who are underperforming, seek to build supportive feedback cultures through system changes and faculty development.

The gaps identified by the review, also alert us to future research implications. A realist review of underperformance and feedback interventions in clinical settings might help build theory and test the utility of SDT as an explanatory theory. The current review is silent on how power is negotiated within feedback encounters especially given the vulnerable position underperforming trainees may hold in relation to their supervisors and assessors. Finally, we suggest that more research is needed to examine how minority and intersecting identities influence feedback conversations about performance and their interpretations.

## 6. Conclusion

Overall, we have presented a view of feedback that is more complex than a process of telling and where professional judgement is needed to work with these educational principles and values. We identified several feedback landscape features required to better support trainees who are underperforming through scaffolding relationality, competence and autonomy. These features included nurturing relationships, fostering productive emotions, enabling the development of feedback literacy (and evaluative judgement) and nurturing supportive feedback cultures. We have argued that we need to shift the dial from an individual deficit-based approach to embracing the complexity of feedback and underperformances in order to support trainees to make sense of and improve how they are progressing in the clinical environment.

## Author contributions

All authors listed have made a substantial, direct, and intellectual contribution to the work and approved it for publication.

## Conflict of interest

The authors declare that the research was conducted in the absence of any commercial or financial relationships that could be construed as a potential conflict of interest.

## Publisher’s note

All claims expressed in this article are solely those of the authors and do not necessarily represent those of their affiliated organizations, or those of the publisher, the editors and the reviewers. Any product that may be evaluated in this article, or claim that may be made by its manufacturer, is not guaranteed or endorsed by the publisher.
